# Engineering xylose utilization in *Yarrowia lipolytica* by understanding its cryptic xylose pathway

**DOI:** 10.1186/s13068-016-0562-6

**Published:** 2016-07-21

**Authors:** Gabriel M. Rodriguez, Murtaza Shabbir Hussain, Lauren Gambill, Difeng Gao, Allison Yaguchi, Mark Blenner

**Affiliations:** Department of Chemical and Biomolecular Engineering, Clemson University, 206 S. Palmetto Blvd., Clemson, SC 29634 USA

**Keywords:** Xylose, *Yarrowia lipolytica*, Cryptic pathway, Metabolic engineering

## Abstract

**Background:**

The oleaginous yeast, *Yarrowia lipolytica*, has been utilized as an industrial host for about 60 years for various applications. Recently, the metabolic engineering of this host has become increasingly popular due to its ability to accumulate lipids as well as improvements made toward developing new genetic tools. *Y. lipolytica* can robustly metabolize glucose, glycerol, and even different lipid classes. However, little is known about its xylose metabolizing capability. Given the desirability of having a robust xylose utilizing strain of *Y. lipolytica*, we performed a comprehensive investigation and elucidation of the existing components of its xylose metabolic pathway.

**Results:**

A quick and efficient means of determining functionality of the candidate xylose pathway genes (XYR, XDH, and XKS) from *Y. lipolytica* was desirable. We challenged *Escherichia coli* mutants lacking either the xylose isomerase (*xylA*) gene or the xylulose kinase (*xylB*) gene to grow on xylose minimal media by expressing the candidate genes from *Y. lipolytica*. We showed that the XKS of *Y. lipolytica* is able to rescue xylose growth of *E. coli* Δ*xylB,* and the XDH enabled growth on xylitol, but not on xylose, of *E. coli* Δ*xylA*. Overexpression of XKS and XDH in *Y. lipolytica* improved growth on xylitol, indicating that expression of the native enzymes was limiting. Overexpression of XKS and XDH in *Y. lipolytica* also enables robust growth on xylose under high nitrogen conditions without the need for adaptation. These results prove that a complete xylose pathway exists in *Y. lipolytica*, but the pathway is poorly expressed. To elucidate the XYR gene, we applied the *E. coli* Δ*xylA* xylose growth challenge with 14 candidate XYR genes and XDH. The XYR2 candidate was able to rescue growth of *E. coli* Δ*xylA* xylose on minimal media.

**Conclusions:**

While a native xylose pathway exists in *Y. lipolytica*, the microorganism’s inability to grow robustly on xylose is an effect of cryptic genetic circuits that control expression of key enzymes in the metabolic pathway. We have characterized the key enzymes associated with xylose metabolism and demonstrated that gene regulatory issues can be overcome using strong hybrid promoters to attain robust growth on xylose without adaptation.

**Electronic supplementary material:**

The online version of this article (doi:10.1186/s13068-016-0562-6) contains supplementary material, which is available to authorized users.

## Background

Metabolic engineering has made significant advancements in the last decade toward engineering microbes that produce a variety of industrially useful products [1]. A large emphasis has been placed on developing downstream metabolic pathways to produce novel products, including alkanes [2], alcohols [3–5], aldehydes [6–8], esters [4, 9], fatty acids [4, 10, 11], and lipid-based products [4, 12] from traditional carbon feedstocks such as glucose. Advancements in lignocellulose degradation have made unconventional sugars abundantly available [13, 14]. The hemicellulose in plant biomass is the third most abundant polysaccharide on earth and is made primarily of xylose, with small amounts of galactose and arabinose. The increasing availability of these alternative sugars has made upstream metabolic engineering for alternative sugar consumption critical to improve economical microbial production of chemicals. As result, much effort has gone toward engineering traditional industrial hosts such as *S. cerevisiae* for efficient xylose consumption [15].

The non-conventional yeast, *Yarrowia lipolytica*, has been utilized as an industrial host for about 60 years for various applications, including single-cell protein and citric acid production [16]. As an oleaginous microorganism, *Y. lipolytica* can naturally accumulate greater than 20 % of its dry cell weight as neutral lipids, typically achieving up to 40–50 % lipid. More recently, this yeast has been engineered to produce high lipid titers and up to 90 % of its dry cell weight as lipids [16, 17]. This innate capacity to produce fatty acids and lipids has sparked growing interest in metabolic engineering of *Y. lipolytica* to produce fatty acid-derived chemicals. These efforts have been enabled by the availability of genome sequence data [18], as well as a growing genetic engineering toolkit, including integration and episomal vectors, finely tuned promoters, and genome editing by CRISPR–Cas9 [19–24]. The naturally high lipid production capabilities of *Y. lipolytica* have been harnessed for the production of lipids [25], omega-3 fatty acids [12], and dicarboxylic acids [26, 27].

**Fig. 1:**
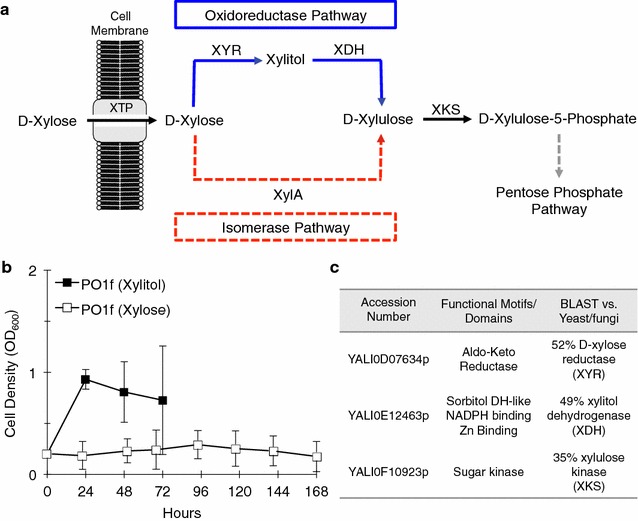
Preliminary assessment of xylose and xylitol metabolism in *Y. lipolytica*. **a** Eukaryotic (*blue*) and prokaryotic (*red*) xylose pathways typical found in nature. The oleaginous yeast, *Y. lipolytica,* is most likely to have a xylose reductase (XYR), xylitol dehydrogenase (XDH), and xylulose kinase (XKS). **b** Growth curve of *Y. lipolytica* PO1f (WT) in xylitol (*black*) and xylose (*white*) YSC media. **c** The top hits from BLAST analysis of the *Y. lipolytica* genome against known yeast xylose metabolism enzymes (XYR, XDH, and XKS). All *error bars* represent SD. (*n* = 3), except for (**b**) (*n* = 4)

*Yarrowia lipolytica* can robustly metabolize glucose, glycerol, alkanes, and even different lipid classes [27]. However, little is known about its xylose metabolizing capability. Most xylose metabolizing yeast, such as *Scheffersomyces stipitis*, utilizes the oxidoreductase pathway; however, *Piromyces* utilizes the isomerase pathway, likely through horizontal gene transfer from bacteria that also commonly use the isomerase pathway (Fig. 1a) [28, 29]. In the literature, there are conflicting reports concerning xylose consumption by *Y. lipolytica* [25, 30–32]. Our results show that *Y. lipolytica* is unable to naturally grow on xylose as the sole carbon source. However, we do observe a small amount of growth on xylitol, an intermediate in the oxidoreductase xylose pathway. This suggests that *Y. lipolytica* contains at least a partially active xylose pathway.

Given the desirability of having a robust xylose utilizing strain of *Y. lipolytica*, we performed a comprehensive investigation and elucidation of the existing components of its xylose metabolic pathway. Initially, we used bioinformatics tools to identify candidate genes that could be responsible for xylose metabolism. Candidate genes were overexpressed in *E. coli* deficient in the xylose genes (*xylA* and *xylB*) to quickly and easily identify positive candidates through in vivo complementation. Active genes were assayed in vitro to confirm activity and then overexpressed in *Y. lipolytica* to observe changes in growth using both xylitol and xylose. Overall, our study shows that *Y. lipolytica* possesses a functional and complete but cryptic metabolic pathway for xylose, and this knowledge was used to engineer robust utilization of xylose.

## Results

### Exploring native xylose and xylitol metabolism

There have been conflicting reports in the literature about the endogenous ability of *Y. lipolytica* to metabolize xylose [25, 30–32]. Our results with *Y. lipolytica* PO1f strain show that the microorganism is unable to utilize xylose as a sole carbon source (Fig. 1b). However, *Y. lipolytica* does exhibit weak growth in D-xylitol (Fig. 1b). Therefore, we first sought to identify whether there were any candidate genes coding for enzymes in the oxido-reductase pathway of xylose metabolism in *Y. lipolytica*. In yeast systems, the oxido-reductase pathway is the major type of metabolic pathway for xylose utilization. Well-characterized xylose metabolism enzymes from *S. stipitis* (XYR: XP_001385181, XDH: XP_001386982, and XKS: XP_001387325) were used as the query to BLAST against the *Y. lipolytica* CLIB 122 protein database. The results of the top hits for each of the hypothetical enzymes, xylose reductase (XYR1), xylitol dehydrogenase (XDH), and xylulose kinase (XKS) are summarized (Fig. 1c).

**Fig. 2:**
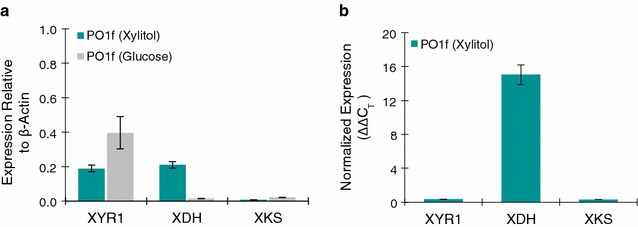
mRNA Quantification of *Y. lipolytica* grown on xylitol. **a** Comparison of mRNA levels of the candidate xylose pathway when grown on xylitol vs glucose. mRNA levels are represented as copy number relative to the copy number of β-actin. **b** Normalized expression using the ∆∆C_T_ method of the candidate xylose metabolism genes when grown on xylitol vs glucose. All error bars represent SD. (*n* = 3)

Since no growth was observed with xylose as the sole carbon source, xylitol was initially used for growth. The native mRNA expression and extent of inducibility (∆∆C_T_) of these three candidate genes were measured using quantitative PCR (Fig. 2a, b). We observed that of the three genes, XDH is the only gene that is induced with xylitol (Fig. 2b). XYR1 is constitutively expressed, while XKS is weakly expressed (Fig. 2a). These results suggest that one of the major bottlenecks for xylose and xylitol metabolism could be the limited expression of XKS.

### *E. coli* growth challenge to test candidate xylose metabolism genes in *Y. lipolytica*

**Fig. 3:**
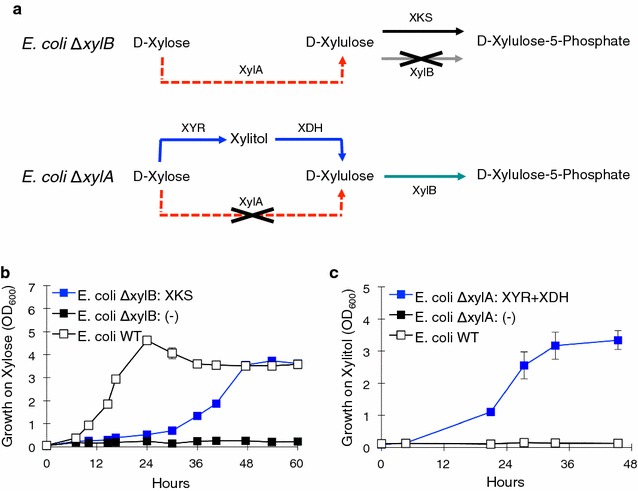
Elucidation of function of XDH and XKS using *E. coli* and further studies in *Y. lipolytica*. **a** Schematic of the xylose growth challenge in *E. coli* using mutants lacking a native gene (*xylA* or *xylB*) in the xylose pathway and challenging *E. coli* to use the candidate *Y. lipolytica* enzyme(s) instead. **b** Growth curves of *E. coli* Δ*xylB* strains in the xylose challenge: *E. coli* Δ*xylB* expressing XKS (Plasmid 2) from *Y. lipolytica* (*blue*), wild-type (BW25113) positive control (*white*), and *E. coli* Δ*xylB* negative control (Plasmid 1) (*black*). **c** Growth curves of *E. coli* Δ*xylA* strains in the 1 % xylitol minimal media: *E. coli* Δ*xylA* expressing XYR1-XDH (Plasmid 3) from *Y. lipolytica* (*blue*), Wild type (BW25113) control (*white*), and *E. coli* Δ*xylB* negative control (Plasmid 1) (*black*). All *error bars* represent SD. (*n* = 3)

Since growth on xylose by *Y. lipolytica* was not observed, attaining further information on the potential xylose metabolic pathway in this host would be difficult and unproductive. A quick, inexpensive, and efficient means of determining if the candidate genes (XYR1, XDH, and XKS) are active was desirable. Instead of using *Y. lipolytica*, we devised a metabolic engineering strategy using *Escherichia coli*. We challenged *E. coli* mutants lacking either the xylose isomerase (*xylA*) gene or the xylulose kinase (*xylB*) gene to grow on xylose minimal M9 media by complementation with the candidate genes from *Y. lipolytica* (Fig. 3a). If the *Y. lipolytica* candidate genes were active toward their expected substrates and expressed functionally in *E. coli,* then they would rescue the growth phenotype of either *E. coli* Δ*xylA* or *E. coli* Δ*xylB*.

First, the *E. coli* BW25113 Δ*xylB* from the Keio collection [33] was acquired and transformed with a plasmid harboring XKS (*Y. lipolytica*) under the pLlacUV5 IPTG inducible promoter. An *E. coli* BW25113 Δ*xylB* harboring a negative plasmid was used as a negative control, and *E. coli* BW25113 wild type was used as a positive control. These strains were inoculated from overnight cultures into M9 media containing 10 g L^−1^ xylose and allowed to grow (Fig. 3b). The *E. coli* Δ*xylB* mutant expressing XKS was found to grow on xylose, while the negative control was unable to grow on xylose. This indicates that the XKS candidate from *Y. lipolytica* is in fact a functional xylulose kinase. In addition, this shows that the *E. coli* xylose challenge allowed for quick and simple confirmation of the candidate enzyme function.

Next, the XYR1 and XDH genes were tested in the *E. coli* xylose challenge using the *E. coli* BW25113 Δ*xylA* acquired from the Keio collection [33]. The *E. coli* Δ*xylA* strain was transformed with a plasmid harboring XYR1 and XDH on an operon under the pLlacUV5 IPTG inducible promoter as before, and the strains were challenged to grow on xylose. Again, a negative control strain and the WT strain were used as a negative and positive control. In this case, we did not observe any growth of the strain harboring the XYR1 and XDH strain in xylose media (data not shown). However, when challenging these strains to grow on xylitol rather than xylose (Fig. 3c), we observed that the strain harboring XYR1 and XDH grows on xylitol, while both the negative and WT controls were unable to grow on xylitol. This proves that the XDH candidate is in fact a functional xylitol dehydrogenase.

The *E. coli* xylose growth challenge proved valuable in quickly confirming the functionality of the XDH and XKS candidates of xylose metabolism enzymes from *Y. lipolytica*. While the XYR1 enzyme did not enable *E. coli* Δ*xylA* to grow in xylose, we cannot rule out the possibility that this enzyme simply does not express functionally in *E. coli*. Nonetheless, the ease and rapidity of these experiments proved valuable.

### Overexpression and knockout studies of XDH and XKS in *Y. lipolytica*

**Fig. 4:**
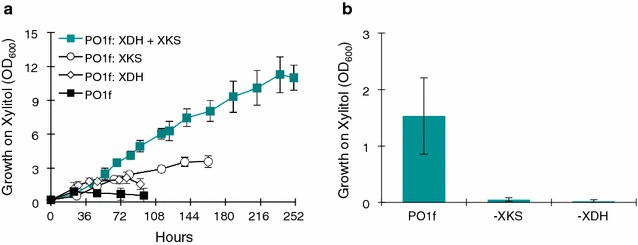
Expression of XDH and XKS improves growth of *Y. lipolytica* on Xylitol. **a** Grow curves of *Y. lipolytica* PO1f strains in 2 % xylitol YSC media while expressing the following genes: XKS (Plasmid 21) (*white circle*), XDH (Plasmid 22) (*white diamond*), both XKS and XDH (Plasmids 21 and 22) (teal), and empty vector control (plasmid 18) (*black*). **b** Cell density (OD_600_) of *Y. lipolytica* PO1f mutants (−XKS or −XDH) in 2 % xylitol YSC media after 96 h: PO1f wild-type control harboring Plasmid 18, −XKS mutant harboring Plasmid 27. −XDH mutant harboring Plasmid 24. All *error bars* represent SD. (*n* = 3)

Having successfully confirmed the activity of the *Y. lipolytica* XDH and XKS gene in the *E. coli* xylose growth challenge experiments, we performed overexpression studies in *Y. lipolytica* to observe the effect of the XDH and XKS genes on xylitol growth. The XDH and XKS genes were expressed under the UAS1B8-TEF_MIN_ synthetic hybrid promoter previously characterized in the literature [20, 21, 24]. We measured the growth of *Y. lipolytica* in YSC media containing 2 % (w/v) xylitol, wherein we expressed both genes together and each gene individually (Fig. 4a). Individually expressing either the XDH or the XKS gene enabled small improvements in the rate of growth. The maximum cell density of the XKS expressing strain was higher (OD_600_ ~3) than the XDH (OD_600_ ~2.15) expressing strain, perhaps indicating that XKS is somewhat more limiting that the XDH. Coexpression of XDH and XKS showed substantial improvement over the individually expressed strains. The strain grew consistently in a linear fashion to reach a final OD_600_ >11, which is nearly 10-fold higher that the wild-type *Y. lipolytica* (OD_600_ ~1.25). The substantial improvement in growth by the XDH/XKS double expression strain suggests that expression of both enzymes are rate limiting. This is in agreement with the qPCR results of the native strain grown on xylitol (Fig. 2a, b), where we observe that the XKS gene has nearly undetectable mRNA levels in xylitol and that the XDH gene is induced by xylitol ~15-fold; however, the expression is still low relative to β-actin expression (~0.2).

Given the improved growth of *Y. lipolytica* on xylitol, we tested the essentiality of these genes for growth on xylitol by conducting gene disruption studies. Here, we applied an early example of the CRISPR–Cas9 system to *Y. lipolytica* [19] to generate a frame shift mutation in the target gene, rendering the protein non-functional. We applied CRISPR–Cas9, as described recently, to disrupt either the XKS or XDH gene in the *Y. lipolytica* genome. These mutants were tested for their ability to grow on xylitol containing YSC media (Fig. 4b). As expected, both the XKS and XDH disrupted mutants were unable to grow on xylitol containing YSC media, demonstrating the essentiality of XDH and XKS genes for the xylose/xylitol metabolism of *Y. lipolytica.*

### Overexpression of XDH and XKS enables *Y. lipolytica* growth on Xylose

**Fig. 5:**
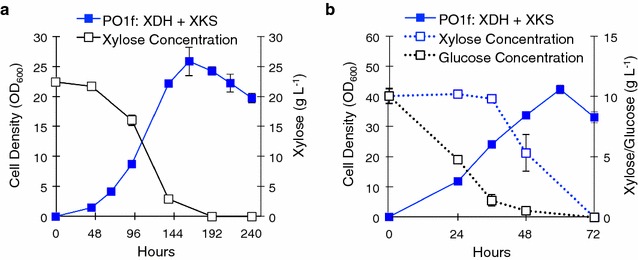
Expression of XDH and XKS enables growth of *Y. lipolytica* on Xylose. **a** Growth curve and xylose consumption of *Y. lipolytica* PO1f expressing XKS and XDH (Plasmids 21 and 22, respectively) in 2 % Xylose YSC media. **b** Growth curve and xylose/glucose consumption of *Y. lipolytica* PO1f expressing XKS and XDH (Plasmids 21 and 22, respectively) in 2 % xylose YSC media. All *error bars* represent SD. (*n* = 3)

The observed functionality of native XDH and XKS gave us the basis to begin engineering efforts to enable *Y. lipolytica* to grow on xylose. All we initially required was the addition of a functional XYR to express along with the *Y. lipolytica* XDH and XKS genes. The introduction a XYR gene into other hosts, such as *S. cerevisiae,* is common and has been demonstrated with several known xylose reductases, most commonly, the XYR of *S. stipitis* [15]. However, while running initial control experiments with the double overexpression of XDH and XKS in *Y. lipolytica*, we found that this strain can indeed grow on xylose as the sole carbon source (Fig. 5a). In fact, the strain was able to reach high cell densities (OD_600_ ~25), comparable with the cell densities achieved with glucose as the sole carbon source. Interestingly, this strain did not require any adaptation period, which is often necessary when engineering yeast to utilize xylose [15]. This strain was able to accomplish twice the cell density in comparison to growth on xylitol as the sole carbon source and a fast time to stationary phase (162 vs 236 h). This surprising result indicated that *Y. lipolytica* in fact contains a functional xylose reductase, and that expression of a heterologous XYR (or any heterologous enzyme) was not necessary to achieve robust xylose utilization. Overexpression of XDH and XKS resulted in nearly identical growth compared to the empty vector transformed wild-type Po1f (Additional file 1), indicating that XDH and XKS only confer a growth advantage in xylose and not in glucose.

Co-utilization studies of the engineered strain with glucose and xylose showed that while *Y. lipolytica* first consumes glucose as the preferred carbon source, rapid utilization of xylose is observed when glucose in the media falls below a critical concentration of ~2 g L^−1^ (Fig. 5b). It is unclear if this lag is due to regulation preventing xylose metabolism, or if it simply results from the lag in gene expression of the overexpressed XDH and XKS genes. There is little xylose consumption during the first 48 h of growth with xylose as the sole carbon, so even if both substrates were being co-utilized, xylose consumption would be insignificant compared to glucose consumption. Furthermore, the growth rate when xylose is being metabolized in the co-utilization experiment is similar to the rate of growth using glucose alone. This suggests that glucose metabolism is preferred, although the strain is then primed for rapid xylose metabolism post-glucose utilization.

### Searching for the missing XYR and enzymatic characterization

**Fig. 6:**
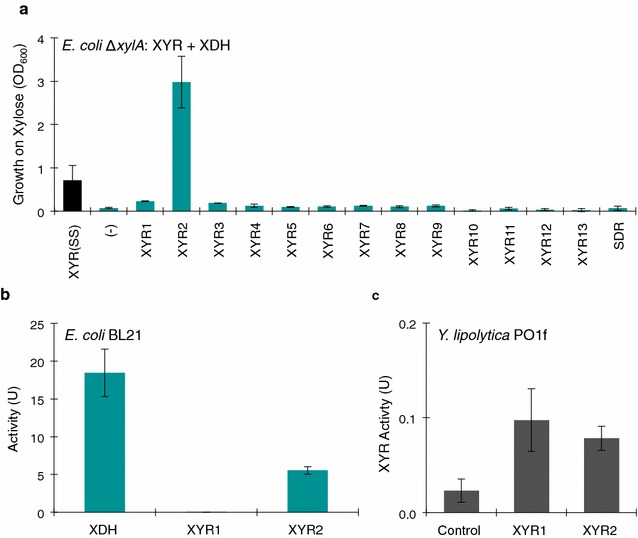
Elucidating XYR genes in *Y. lipolytica* and enzyme characterization. **a** Screening for the missing XYR gene from *Y. lipolytica* by revisiting the *E. coli* xylose challenge. Cell densities of *E. coli* Δ*xylA* strains grown in 1 % xylose minimal media for 6 days expressing a candidate XYR (1–13, or SDR) and XDH (Plasmids 3–16) from *Y. lipolytica* (*teal*) were measured. The known *S. stipitis* XYR (*black*) and XDH (*Y. lipolytica*) (Plasmid 17) were used as a positive control. **b** Enzyme assay of XDH in BL21 cell lysates using NAD^+^ cofactor and xylitol as a substrate. One unit is defined as the reduction of 1 μmol NAD^+^ per min per mg protein. Enzyme assay of XYR1 and XYR2 in BL21 cell lysates using NADPH cofactor and xylose as a substrate. One unit is defined as the oxidation of 1 μmol NADPH per min per mg protein. Protein concentrations were calculated by densitometry from the coomassie image after SDS–PAGE electrophoresis. **c** Enzyme assay of XYR1 and XYR2 in *Y. lipolytica* cell lysates using NADPH cofactor and xylose as a substrate. XYR1 and XYR2 overexpressed using Plasmids 32 and 33. Control strain harboring plasmid 18 was also tested. One unit is defined as the oxidation of 1 μmol NADPH per min per mg protein. Protein concentrations are calculated by Bradford Assay for total cellular protein. All *error bars* represent SD. (*n* = 3)

To find the missing XYR gene, we revisited our original BLAST analysis of xylose reductase and reapplied the *E. coli* xylose growth challenge. Since the growth challenge successfully confirmed the activity of XDH and XKS, there was justification to validate the XYR gene using the same methods. We screened 13 XYR candidates as well as a short chain dehydrogenase/reductase (SDR) from *Y. lipolytica*, known to act on fructose [34] (Additional file 2). Each of the 14 genes was expressed along with XDH (YALI0E12463g) as a single operon. Each plasmid was introduced into *E. coli* Δ*xylA*, and the strains were challenged to grow on xylose M9 media. As a positive control, the XYR gene from *S. stipitis* was tested in the xylose challenge. In addition, the strains were allowed to grow for a considerably longer time. After the 6th day, rapid growth was observed from the XYR2 gene (YALI0F18590g), and some growth was observed from the XYR of *S. stipitis* (Fig. 6a). The prolonged time to observe growth could be due to redox imbalances innate to *E. coli*, but nevertheless demonstrates XYR2 is a functional xylose reductase.

With a complete xylose pathway now elucidated, we expressed the XYR1, XYR2, and XDH in *E. coli* BL21 cells for enzymatic characterization. Crude cell lysates were prepared, and activities were measured with their respective substrate and NAD(P)H cofactors. Activity from XYR2 and XDH was detected, but no activity from XYR1 was detected (Fig. 6b). The XDH enzyme was found to be highly active (~18.5 μg min^−1^ mg^−1^) although mostly insoluble in *E. coli* (Additional file 3). The XYR1 enzyme was found to be insoluble in *E. coli,* and no activity was measured in *E. coli* lysates. Since XYR1 was insoluble, this result does not necessarily reflect a lack of activity in *Y. lipolytica*. The XYR2 enzyme, however, was found to be soluble, and its activity was estimated to be ~5–6 μg min^−1^ mg^−1^, about threefold lower than the XDH enzyme. *E. coli* lysate activity was normalized to specific protein determined by SDS–PAGE densitometry.

We overexpressed XYR1 and XYR2 in *Y. lipolytica* to ascertain the activity of the reductases in their native environment. Strains were grown in 2 % oleic acid YSC media with the appropriate drop out mixture to achieve maximum expression of the synthetic hybrid promoter. Cell lysates were prepared and assayed for XYR activity (Fig. 6c). A control strain harboring an empty vector was also assayed. Both XYR1 and XYR2 showed statistically similar amounts of activity (0.08–0.1 U), indicating that both XYR1 and XYR2 are functional reductases. The control strain showed small amounts of activity likely due to the constitutive expression of the XYRs as determined by qPCR (Fig. 2a). *Y. lipolytica* lysate activity was normalized to total protein determined by a Bradford assay.

### qPCR analysis of the xylose utilizing *Y. lipolytica* strain

**Fig. 7:**
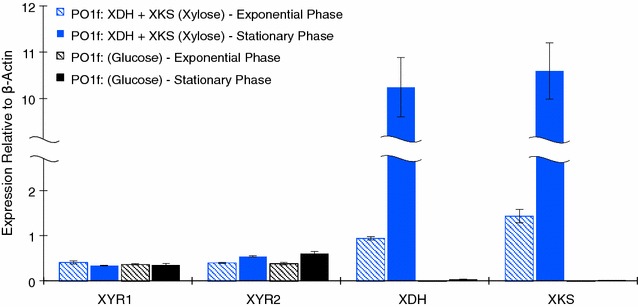
mRNA quantification of the xylose utilizing *Y. lipolytica* strain. **a** Early and late exponential phase measurement of mRNA copy number relative to β-actin mRNA copy number for the xylose pathway in *Y. lipolytica* PO1f expressing XKS and XDH (Plasmids 21 and 22, respectively) (*blue*) grown in 2 % xylose YSC–LEU–URA media compared to *Y. lipolytica* PO1f harboring Plasmids 18 (*empty vectors*) (*black*) grown in 2 % glucose YSC-LEU–URA. All *error bars* represent SD. (*n* = 3)

We compared mRNA levels of four xylose metabolism genes (XYR1, XYR2, XDH, and XKS) from the engineered XDH + XKS strain and the wild-type PO1f. mRNA expression relative to a reference gene (β-actin) was measured by qPCR at early exponential phase and at late exponential/early stationary phase, respectively. We found that in either phase of growth, expression levels of XYR1 and XYR2 genes are always similar to expression levels of β-actin, indicating constitutive expression. This observation is consistent across the XDH + XKS strain and the wild type. Expression of the XDH and XKS genes under the control of the UAS1B8-TEF_MIN_ promoter have similar transcriptional levels compared to β-actin (0.9 and 1.4, respectively) in early exponential phase, but expression increases ~10-fold in stationary phase (10.2 and 10.6, respectively) (Fig. 7). This increase in expression during late exponential phase is characteristic of this hybrid promoter system [21, 24].

### Lipid analysis of the engineered xylose utilizing *Y. lipolytica*

**Fig. 8:**
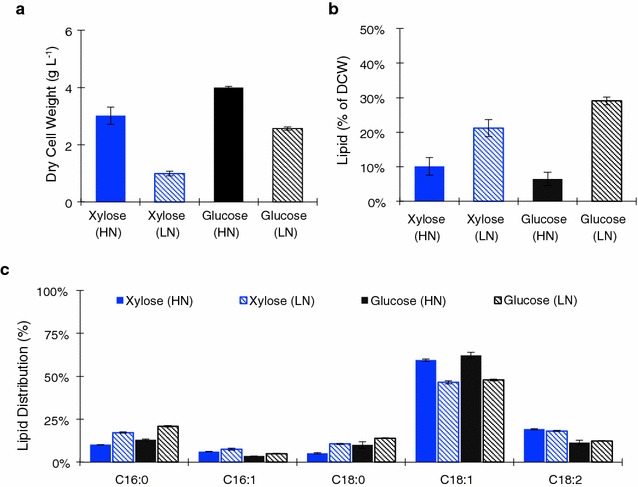
Dry Cell weight and lipid analysis of the xylose utilizing strain as compared to glucose. **a** Dry cell weight (DCW) measurement of *Y. lipolytica* PO1f harboring Plasmids 21 and 22 (XKS and XDH) grown in high and low nitrogen media containing 2 % xylose and *Y. lipolytica* PO1f harboring Plasmid 18 (*empty vector*) grown in high and low nitrogen media containing 2 % glucose: xylose and high nitrogen (*blue*), xylose and low nitrogen (*striped blue*), Glucose and high nitrogen (*black*), glucose and low nitrogen (*striped black*). **b** Lipid content as a percentage of DCW for the strains and culture conditions listed above. **c** Fatty acid profile as a percentage of total lipids for the strains and culture conditions listed above: xylose and high nitrogen (*blue*), xylose and low nitrogen (*striped blue*), glucose and high nitrogen (*black*), glucose and low nitrogen (*striped black*). All *error bars* represent SD. (*n* = 3)

A comparison of lipid production and the lipid profile was made between the engineered xylose strain grown on xylose and wild-type (PO1f) grown on glucose. High and low nitrogen conditions were tested (Fig. 8a–c). Under high nitrogen conditions, the xylose strain reached a dry cell weight nearly as large as the wild type grown on glucose (3 vs 4 g L^−1^), while accumulating a higher percentage of lipids (10 vs 6 % of DCW). In low nitrogen conditions, the xylose strain performed less well in terms of dry cell weight as compared with wild type (1 vs 3 g L^−1^), and accumulated slightly less lipids as percentage of dry cell weight (21 vs 29 %).

Compared with high nitrogen conditions, overall lower final cell mass accumulation and growth rates were observed under low nitrogen for the engineered strain (grown on xylose) compared to the wild type (grown on glucose). This is a result of a global regulatory process known to occur in *Y. lipolytica* where there is a shift from cell growth to lipid accumulation during cell maturation under nitrogen limitation. However, a bigger difference in dry cell weight is observed between the two nitrogen conditions with engineered strain (~66 %) compared with the wild-type strain (~25 %). This could be a combinatorial effect of using hybrid promoters that elicit strongest expression strength towards late exponential and stationary phase and the lack of adaptation of the engineered strain, which may contribute to improved growth and lipid yields. Undoubtedly, there exist some bottlenecks associated with the initial xylose utilizing strain when compared to glucose utilization that need to be addressed in the future studies.

## Discussion

In this study, we have expanded the substrates utilized by *Y. lipolytica* to include xylose. We described a cryptic xylose metabolism pathway in *Y. lipolytica*, and elucidated the full set of enzymes responsible for the conflicting reports in the literature with regard to its xylose utilization capabilities. To provide a more complete understanding of the innate capabilities of *Y. lipolytica* for xylose metabolism, we have studied the biochemical and metabolic roles of predicted xylose metabolism enzymes and concluded that XYR1, XYR2, XDH, and XKS are functional, and that XDH and XKS are required for xylose metabolism. Using *E. coli* complementation assays and measurements in *Y. lipolytica* with xylitol as a substrate, we identified the XDH and XKS steps as key bottlenecks in xylose metabolism. We also identified the essentiality of these enzymes in xylitol metabolism. Furthermore, we found that overexpression of XDH and XKS resulted in robust growth on xylose, reaching an OD_600_ of 25 after 7 days. This is approximately three times the cell titer shown by Ryu et al. in a recent study on xylose metabolism that was published, while we prepared this manuscript [35]. Finally, we showed that the lipids produced by *Y. lipolytica* with overexpression of XDH and XK were nearly identical in composition to those from glucose and yielded similar lipid mass.

By applying a xylose growth challenge strategy using *E. coli* mutants lacking a native xylose gene (*xylA* or *xylB*) and forcing *E. coli* to utilize the candidate *Y. lipolytica* enzymes, we were able to quickly confirm the XDH and XKS enzyme in the xylose pathway. The expression of XDH and XKS (*Y. lipolytica*) allowed the *E. coli* mutants to grow on xylitol and xylose, respectively. To find the missing XYR gene, 14 candidate XYR genes were tested in the *E. coli* Δ*xylA* growth challenge. The XYR2 gene was the only candidate gene that enabled *E. coli* Δ*xylA* to grow on xylose. XYR2 enabled faster growth to higher titers than XYR from *S. stipitis* in the *E. coli* growth challenge. Enzymatic characterization of XYR2 and XDH in *E. coli* lysates showed robust activity from both enzymes. No activity was detected from XYR1, but this enzyme was found to be insoluble in *E. coli,* so we could not draw a definitive conclusion about the relative activities of these two enzymes. However, overexpression of the XYR1 or XYR2 gene in *Y. lipolytica* results in similar levels of xylose reductase activity.

We found that XYR1 and XYR2 are constitutively expressed in *Y. lipolytica* and do not undergo carbon catabolite repression in glucose; however, mRNA expression is still low compared to β-actin. Activity assays of XYR1 and XYR2 overexpressed in *Y. lipolytica* show statistically equal activity. We have definitively shown that XYR2 has activity approximately equal to that measured for XYR1 by Ryu et al. and that mRNA expression of these two genes is approximately equal [35]. We suggest that XYR1 and XYR2 may serve redundant roles.

qPCR analysis of the wild type grown on xylitol as compared to glucose showed inducibility of the XDH gene and no inducibility of the XKS gene. In strains where we overexpress XDH and XKS, we observe significant mRNA copy number driven by the strong hybrid promoters used. We do not observe any xylose inducible expression from either XYR1 or XYR2. Ryu et al. measured relative mRNA using their xylose adapted wild-type *Y. lipolytica* and saw mild inducibility of all three genes [35]. In this work, we measured mRNA levels using gene specific calibration curves instead of simply using the ∆∆Ct method to determine the extent to which genes were being expressed. Overall, the expression level is low relative to the house keeping gene, β-actin. Strictly going by expression level, XKS is expressed the weakest implying it is a possible bottleneck in xylose metabolism. By overexpressing XDH and XKS genes in *Y. lipolytica,* we showed that the double overexpression strain grew to ~10-fold higher cell density (OD_600_ >10) on xylitol compared to the wild type. Expressing XKS alone yielded better growth than expressing XDH alone, which is in agreement with the qPCR results. These results indicated that native expression of both XDH and XKS is insufficient to achieve robust grow on xylitol.

Overexpressing XDH and XKS genes in *Y. lipolytica* also enabled robust growth on xylose as the sole carbon source without the need for adaptation. This surprising result proved that *Y. lipolytica* contains a complete xylose pathway including functional XYRs, which was not originally identified when we only examined XYR1. Disruption of the XDH and XKS genes using a recently developed CRISPR–Cas9 system [19] showed that both genes are essential for growth on xylitol.

Our approach of activating the cryptic xylose metabolism by overexpression of rate limiting enzymes yielded a xylose metabolizing strain that remarkably did not require any adaptation. Ryu et al. showed that the wild-type strain requires adaptation to get relatively subtle growth on xylose [35]. Likewise, Tai showed that when the oxido-reductase pathway was engineered into *Y. lipolytica* adaptation was also required [32]. The explanation for what occurs during this adaptation remains an active area of inquiry.

Interestingly, in co-utilization experiments, we observe a much more rapid depletion of xylose, suggesting that the *Y. lipolytica* is capable of rapid xylose metabolism if additional bottlenecks are addressed. Rate of growth on xylose as a sole carbon source was still was slower than growth on glucose (168 vs ~48 h), indicating that further bottlenecks exist. Potential bottlenecks for xylose metabolism are well appreciated and include limitations in transporter activity/expression, redox balance, enzyme activity, downstream pentose phosphate enzymes, and due to the nature of the hybrid promoters used in this study where expression is several folds lower in early exponential phase as compared to late exponential phase.

## Conclusions

We have elucidated the native pathway for xylose metabolism in *Y. lipolytica*. The microorganism’s inability to grow robustly on xylose is the result of insufficient activation of native xylose metabolism genes. In this publication, we have characterized the key enzymes associated with xylose metabolism and demonstrated that gene regulatory issues can be overcome using strong hybrid promoters to attain robust growth on xylose without adaptation. Constitutive overexpression of the cryptic XKS gene and weakly inducible XDH gene can result in growth to high cell densities. We also note comparable lipid accumulation profiles under high nitrogen conditions for strains metabolizing glucose and xylose as a sole carbon source, respectively. Under low nitrogen conditions, however, the lipid yields are somewhat diminished in the xylose metabolizing strain using xylose as the sole carbon source. Improvements to growth rates and lipid accumulation can be attained by the development of better genetic tools that confer strong, constitutive gene expression to key genes early on during growth. Furthermore, improving the flux of xylose into the cell via the use of xylose-specific transporters could aid in more efficient uptake for metabolism.

## Methods

### Reagents

All chemicals were purchased from Sigma unless otherwise stated. All restriction enzymes, DNA ligases, and DNA polymerases used for cloning and PCR were purchased from New England Biolabs (NEB) unless otherwise stated. Plasmid minipreps, PCR purifications, and gel extractions were done using the Zymo Zyppy Plasmid Miniprep Kit, and Zymo DNA Clean and Concentrator 5. Genomic DNA from *Y. lipolytica* was extracted using the E.Z.N.A. Yeast DNA kit (Omega Biotek). All oligonucleotides and gBlocks^®^ were purchased from IDTDNA. Xylose assays were performed using the D-Xylose Assay Kit (Megazyme). Glucose assays were done using glucose oxidase and horseradish peroxidase enzymes purchased from Sigma.

### *E. coli* strains and culture conditions

The strains used in this study are summarized in Table 1. *Escherichia coli* DH10β cells from New England Biolabs (NEB) (Ipsich, MA) were used for cloning and propagation of plasmids in Luria–Bertani (LB) media supplemented with 200 μg mL^−1^ ampicillin. *E. coli* BW25113 Δ*xylA* and Δ*xylB* were obtained from Coli Genetic Stock Center (CGSC) at Yale University (New Haven, CT). *E. coli* BL21 (DE3) was used as the host organism to study functional xylose genes of *Y. lipolytica*.

**Table 1:** Strains and plasmids

	Genotype	References
*E. coli* strains		
BW25113	*rrnB*_T14_ Δ*lacZ*_WJ16_ hsdR514 Δ*araBAD*_AH33_ Δ*rhaBAD*_LC78_	[33, 39]
BW25113 Δ*xylA*	BW25113 with *xylA::kanR*	[33, 39]
BW25113 Δ*xylB*	BW25113 with *xylB::kanR*	[33, 39]
BL21(DE3)	*fhuA2 [lon] ompT gal (λ DE3) [dcm] ∆hsdS* *λ DE3* = *λ sBamHIo ∆EcoRI*-*B int::(lacI::PlacUV5::T7* *gene1) i21 ∆nin5*	[40]
*Y. lipolytica* strains		
P01f	*MatA, leu2*-*270, ura3*-*302, xpr2*-*322, axp1*-*2* (ATCC no. MYA-2613)	ATCC
P01f–XKS	P01f with alternate frameshift mutation in XKS gene	This work
P01f–XDH	P01f with frameshift mutation in XDH gene	This work
Plasmids		
1	Amp^R^, ColE1 ori; P_lacUV5_: fadD-AtfA, *lacI* ^*q*^	[4]
2	Amp^R^, ColE1 ori; P_lacUV5_: XKS (YALI0F10923 g), *lacI* ^*q*^	This work
3	Amp^R^, ColE1 ori; P_lacUV5_: XYR1 (YALI0D07634 g)-XDH (YALI0E12463 g), *lacI* ^*q*^	This work
4	Amp^R^, ColE1 ori; P_lacUV5_: XYR2 (YALI0F18590 g)-XDH (YALI0E12463 g), *lacI* ^*q*^	This work
5	Amp^R^, ColE1 ori; P_lacUV5_: XYR3 (YALI0A15906 g)-XDH (YALI0E12463 g), *lacI* ^*q*^	This work
6	Amp^R^, ColE1 ori; P_lacUV5_: XYR4 (YALI0B21780 g)-XDH (YALI0E12463 g), *lacI* ^*q*^	This work
7	Amp^R^, ColE1 ori; P_lacUV5_: XYR5 (YALI0C13508 g)-XDH (YALI0E12463 g), *lacI* ^*q*^	This work
8	Amp^R^, ColE1 ori; P_lacUV5_: XYR6 (YALI0B07117 g)-XDH (YALI0E12463 g), *lacI* ^*q*^	This work
9	Amp^R^, ColE1 ori; P_lacUV5_: XYR7 (YALI0E18348 g)-XDH (YALI0E12463 g), *lacI* ^*q*^	This work
10	Amp^R^, ColE1 ori; P_lacUV5_: XYR8 (YALI0042092 g)-XDH (YALI0E12463 g), *lacI* ^*q*^	This work
11	Amp^R^, ColE1 ori; P_lacUV5_: XYR9 (YALI0C09119 g)-XDH (YALI0E12463 g), *lacI* ^*q*^	This work
12	Amp^R^, ColE1 ori; P_lacUV5_: XYR10 (YALI0F06974 g)-XDH (YALI0E12463 g), lacI^q^	This work
13	Amp^R^, ColE1 ori; P_lacUV5_: XYR11 (YALI0B15268 g)-XDH (YALI0E12463 g), *lacI* ^*q*^	This work
14	Amp^R^, ColE1 ori; P_lacUV5_: XYR12 (YALI0C00319 g)-XDH (YALI0E12463 g), *lacI* ^*q*^	This work
15	Amp^R^, ColE1 ori; P_lacUV5_: XYR13 (YALI0A19910 g)-XDH (YALI0E12463 g), *lacI* ^*q*^	This work
16	Amp^R^, ColE1 ori; P_lacUV5_: SDR (YALI0D18964 g)-XDH (YALI0E12463 g), *lacI* ^*q*^	This work
17	Amp^R^, ColE1 ori; P_lacUV5_: XYR (XP_001385181)-XDH (YALI0E12463 g), *lacI* ^*q*^	This work
18	Amp^R^, ColE1 ori, LEU2, CEN; UAS1B8-TEF-(empty)-CYC1	[21]
19	Amp^R^, ColE1 ori, LEU2, CEN; UAS1B8-TEF-hrGFP-CYC1	[21]
20	Amp^R^, ColE1 ori, URA3, CEN; UAS1B8-TEF-hrGFP-CYC1	[21]
21	Amp^R^, ColE1 ori, LEU2, CEN; UAS1B8-TEF-XKS (YALI0F10923 g)-CYC1	This work
22	Amp^R^, ColE1 ori, URA3, CEN; UAS1B8-TEF-XDH (YALI0E12463 g)-CYC1	This work
23	Amp^R^, ColE1 ori, LEU2, CEN; UAS1B8-TEF-cas9-CYC1, TEF-HH-sgRNA-HDV	[19]
24	Amp^R^, ColE1 ori, LEU2, CEN; UAS1B8-TEF-cas9-CYC1, TEF-HH-(XDH)sgRNA-HDV	This work
25	Amp^R^, ColE1 ori, LEU2, CEN; UAS1B8-TEF-cas9-CYC1, SCRp’-tRNAp- (PEX10)sgRNA	[19]
27	Amp^R^, ColE1 ori, LEU2, CEN; UAS1B8-TEF-cas9-CYC1, SCRp’-tRNAp-(XKS)sgRNA	This work
28	Amp^R^, ColE1ori; P_T7lac_: empty, *lacI* ^*q*^ (pETDuet)	Novagen
29	Amp^R^, ColE1ori; P_T7lac_: 6xhis-XDH (YALI0E12463 g), *lacI* ^*q*^	This work
30	Amp^R^, ColE1ori; P_T7lac_: 6xhis-XYR1 (YALI0D07634 g), *lacI* ^*q*^	This work
31	Amp^R^, ColE1ori; P_T7lac_: 6xhis-XYR2 (YALI0F18590 g), *lacI* ^*q*^	This work
32	Amp^R^, ColE1 ori, LEU2, CEN; UAS1B8-TEF-XYR1-CYC1	This work
33	Amp^R^, ColE1 ori, URA3, CEN; UAS1B8-TEF-XYR2-CYC1	This work

### Transformation of *Y. lipolytica* and culture conditions

*Yarrowia lipolytica* strain PO1f (*MATaleu2*-*270 ura3*-*302 xpr2*-*322 axp1*) (ATCC no. MYA-2613) was used for gene deletion, overexpression and growth studies. Transformations of *Y. lipolytica* were performed using the lithium acetate method [23]. General *Y. lipolytica* growth was performed in rich YPD. Growth of transformants was done in yeast synthetic complete (YSC) media that contained 6.7 g L^−1^ yeast nitrogen base (YNB) from MP Biomedicals (Santa Ana, CA) and complete synthetic media with the desired drop out (0.69 g L^−1^ CSM-LEU, 0.77 g L^−1^ CSM-URA or 0.67 g L^−1^ CSM-LEU-URA) purchased from Sunrise Science Products (San Diego, CA). Carbon sources used for the following experiments were either 2 % (w/v) d-glucose from Sigma Aldrich (St. Louis, MO), d-xylitol and d-xylose from Alfa Aesar (Haverhill, MA). Agar plates were made by adding 15 g L^−1^ agar. *Y. lipolytica* growth experiments were first pre-cultured in 2-mL culture tubes prior to inoculating 15-mL cultures in 50 -mL baffled flasks and cultivated at 28 °C and 215 rpm. Low nitrogen conditions were identical to high nitrogen media except ammonium sulfate was omitted from the media.

### Identification of xylose metabolism genes

Putative xylose metabolism genes from *Y. lipolytica* were identified using *S. stipitis* genes for xylose reductase (NCBI: XP_001385181), xylitol dehydrogenase (NCBI: XP_001386982), and xylulose kinase (NCBI: XP_001387325) as the query. The BLAST search [36] was targeted to the *Y. lipolytica* CLIB 122 genome and individual gene DNA sequences were compared to the PO1f genome sequence.

### Plasmid construction

*Yarrowia lipolytica* expression cassettes were created from the base vector pSL16-UAS1B8-TEF(MIN)-hrGFP-cyc1t harboring either the LEU2 or URA3 selective marker that has been previously synthesized and characterized under different carbon conditions [21, 24]. Basic cassettes for gene knockout using CRISPR–Cas9 were obtained from Schwartz et al. [19]. All plasmids (Table 1) were cloned using sequence and ligation-independent cloning (SLIC) [9, 37], except for Plasmids 28 and 29, which were constructed using Gibson Assembly [38]. Oligonucleotides and gBlocks^®^ used are listed in Additional file 4. The target gene(s) and vector fragments were amplified with primer pairs from the templates listed in Additional file 5. In some cases, vector fragments were prepared by digestion with restriction enzymes. The resulting fragments were purified and combined using SLIC. In summary, a 10 μL reaction containing 1 × NEB Buffer 2, 100–1000 ng of vector and insert fragments, and 0.25 μL (0.75 U) of T4 DNA Polymerase (NEB) was incubated at room temperature for 5–10 min, annd then placed on ice to stop the reaction. 2.5 μL of the solution was used for transformation of *E. coli*. Plasmids were verified by digestion with restriction enzymes and by DNA sequencing.

### Xylose and xylitol growth challenge in *E. coli*

*Escherichia coli* BW25113 Δ*xylB* was transformed with Plasmids 1 and 2. *E. coli* BW25113 Δ*xylA* was transformed with Plasmids 1 and 3–17. *E. coli* BW25113 was transformed with Plasmid 1. Colonies were inoculated into culture tubes containing 1 mL M9 minimal media containing 10 g L^−1^ xylose with the appropriate antibiotics and 1 mM IPTG. The strains were allowed to grow at 28 °C, 215 RPM, for up 144 h. To test the XDH activity, xylitol was used instead of xylose.

### Protein expression and lysate preparation

BL21(DE3) was transformed with Plasmids 29–31. Three colonies of each strain were inoculated into each culture tubes containing 3-mL LB with the appropriate antibiotics. Triplicate cultures were made for each strain. The overnight cultures were inoculated by 100-fold dilution into culture tubes containing 3-mL LB media with the appropriate antibiotics. The cultures were allowed to grow at 37 °C, 250 rpm, until an OD_600_ of ~0.4 then induced with 1-mM IPTG. Cultures were allowed to express protein for 3–4 h at 28 °C, 215 rpm. Cultures were then centrifuged at 3000×*g* for 10 min. The media was discarded, and cell pellets were concentrated 10× (300 μL) in 100 mM PBS buffer and 1× BugBuster reagent, and transferred to 1.5-mL tubes. Lysis was allowed to occur for 20 min at 28 °C with shaking (215 RPM). Lysates were then centrifuged at 15,000 rpm and 4 °C, for 10 min. Soluble fractions were transferred to new chilled 1.5-mL tubes. Insoluble fractions were suspended in 100 mM PBS buffer.

For *Y. lipolytica* lysates, strains harboring either plasmid 18 (empty), 32 (XYR1), and 33 (XYR2) were grown were grown in 50-mL baffled flasks containing 15 mL of 2 % oleic acid YSC media with the appropriate drop out mix. Cultures were harvested in stationary phase for optimal protein expression. 15-mL cultures were washed twice in 100 mM PBS containing 5 % Tween80 prior to being harvested for lysis using the YeastBuster™ Protein Extraction Reagent from EMD Millipore (Billerica, MA). Protease inhibitors from Research Products International (RPI) (Mt. Prospect, IL) were added into the cell lysates to prevent protein degradation.

### Enzyme assays of XDH, XYR1, and XYR2

Enzyme assay were performed at 28 °C in 96 well plate format using an Epoch 2 Microplate Spectrophotometer (BioTek). Assay conditions were as follows: 40 μL 100 mM PBS buffer mixed with 10 μL lysates, 100 μL of master mix (86 μL ddH_2_O, 10 μL 1 M PBS buffer, and 4 μL of either 10 mM NADPH or NAD^+^), and 50 μL of either 1 % xylose, 100 mM xylitol, or ddH_2_O (blank). For XYR1 and XYR2 assays, the oxidation of NADPH was followed for 5 min at 340 nm. For XDH assays, the reduction of NAD^+^ was followed for 5 min at 340 nm. One unit of activity is defined as the oxidation of 1 μmol of NADPH or reduction of 1 μmol of NAD^+^ per minute per milligram of protein. For *E. coli* lysate experiments, specific protein concentrations were determined by densitometry of specific protein in SDS–PAGE. For *Y. lipolytica* lysates, protein normalization was to total cellular protein determined by a Bradford assay.

### Quantitative reverse transcription PCR

*Yarrowia lipolytica* transformants grown under different carbon conditions were subject to RNA extraction during early exponential phase and late exponential phase. Prior to RNA extractions, all cultures were normalized to an OD_600_ of 10. For total RNA extraction, the cells were spun down at 6000×*g* for 2 min and normalized to and OD_600_ of 10. RNA was extracted using the E.Z. N.A. Yeast RNA kit. RNA extracts were stored at −80 °C.

Prior to performing any quantitative PCR (qPCR) experiments, qPCR primers for β-actin, TEF, XYR1, XYR2, XDH, and XKS (Additional file 4, Oligos 57–64) were tested for efficiency; only primer pairs with priming efficiencies between 90–110 % were used for subsequent qPCR experiments. A two-step protocol was employed for these experiments. 500 ng of total RNA was used to make cDNA with the Oligo-dT priming cDNA synthesis kit from Thermo Scientific (Waltham, MA). A volume of 1.5 μL of the cDNA synthesis mix was used for each qPCR experiment using the Maxima SYBR Green/Fluorescein qPCR master mix purchased from Thermo Scientific. All experiments were performed in biological triplicates in 96 well plates using the CFX Connect Real-Time Thermocycler (Bio-Rad). Two reference genes (β-actin and TEF) were used to validate trends in expression.

For qPCR, copy numbers for the expression of each of the genes described above were calculated from their respective gene amplification standard curves. The copy number of each gene was normalized against the copy number of the reference gene within the same sample to account for any discrepancies in either RNA or cDNA loading during the experiments. Relative qPCR was performed to observe any trends in inducibility of genes in xylose metabolism. The control sample employed for these experiments was transformants that were grown in glucose to contrast against the experimental samples that were grown in d-xylitol or d-xylose. Priming efficiencies were considered when analyzing normalized expression values for each of the xylose metabolizing genes.

### Glucose and xylose assays

Glucose and xylose assays were performed in pH 7.0 sodium phosphate buffer made from sodium phosphate dibasic and sodium phosphate monohydrate salts. 50-μL culture samples were removed from the culture every two days, centrifuged at 12,000×*g* for 4 min, and 40 μL of supernatant was stored at −20 °C. Samples for xylose assay were diluted 1:20 with YSC-LEU-URA media. Hexokinase treatment even though no glucose was used. XDH/XMR enzyme was diluted 1:4 prior to treatment. The Epoch 2 Microplate Spectrophotometer (BioTek) was used to measure endpoint reaction absorbance. The kinetic assay was run at 25 °C with absorbance measurements taken every minute for 1 h at 340 nm with continuous orbital shaking.

### Dry cell weight and lipid analysis

10 mL of cell culture was washed with ddH_2_O three times and dried overnight at 104 °C. Dry cell weight was measured using an analytical balance. To identify and quantify lipids in cell biomass, lipids were transesterified to FAMEs as described previously [17]. Briefly, 1-mL cell culture was washed with ddH_2_O. 100 μL glyceryl triheptadecanoate (2 mg μL^−1^ methanol) was added to the cell pellet and internal standard. Lipids were transesterified to FAMEs with 500 μL of 0.5 N sodium methoxide (20 g L^−1^ sodium hydroxide in methanol) followed by 40 min of vortexing at 2000 rpm. The solution was then neutralized with 40 μL sulfuric acid. FAMEs were extracted by adding 850 μL hexane followed by 20 min vortexing at 2000 rpm. The mixture was centrifuged for 1 min at 8000 rpm and the hexane layer (750 μL) was collected for GC–FID analysis (Agilent 7890B). Samples were injected with an injection volume of 1 μL, split ratio of 10, and injector temperature of 250 °C. FAME species were separated on an Agilent J&W DB-23 capillary column (30 m × 0.25 mm × 0.15 μm), with helium carrier gas at a flow rate of 1 mL min^−1^. The temperature of the oven started from 175 °C, held for 1 min, and then ramped to 200 °C within 5 min. The FID was operated at a temperature of 280 °C with a helium make up gas flow of 25 mL min^−1^, hydrogen flow of 30 mL min^−1^, and air flow of 300 mL min^−1^.

## Additional files

10.1186/s13068-016-0562-6 Overexpression of XDH and XKS do not confer a growth advantage in glucose media. Growth curves of XDH-XKS strain and empty vector in 2 % glucose.
10.1186/s13068-016-0562-6 Candidate XYR genes in* Y. lipolytica* by BLAST analysis. A list of the putative XYR genes that were screened in this study, with their NCBI accession numbers and their % identity to S. stipitis XYR.
10.1186/s13068-016-0562-6 SDS-PAGE of XDH, XYR1, and XYR2 in *E. coli* BL21 lysates. SDS-PAGE data showing the abundance of insoluble and soluble protein from *E. coli* overexpression of the following enzymes: XDH, XYR1 or XYR2.
10.1186/s13068-016-0562-6 Oligonucleotides used in this study. A complete list of all of the oligonucleotides we used throughout this manuscript.
10.1186/s13068-016-0562-6 SLIC cloning table. A complete list of all plasmids and the construction strategy we used throughout this manuscript.

## Abbreviations

XYR: xylose reductase
XDH: xylitol dehydrogenase
XKS: xylulose kinase
qPCR: quantitative polymerase chain reaction
FID: flame ionization detector
