# Supplementary material for: Engineering xylose utilization in Yarrowia lipolytica by understanding its cryptic xylose pathway
Source: Biotechnol Biofuels. 2016 Jul 21;9:149. doi: 10.1186/s13068-016-0562-6 (PMC4955270; doi:10.1186/s13068-016-0562-6)
Supplement: Supplementary file 1 — 10.1186/s13068-016-0562-6 Overexpression of XDH and XKS do not confer a growth advantage in glucose media. Growth curves of XDH-XKS strain and empty vector in 2 % glucose. [file 13068_2016_562_MOESM1_ESM.pdf]

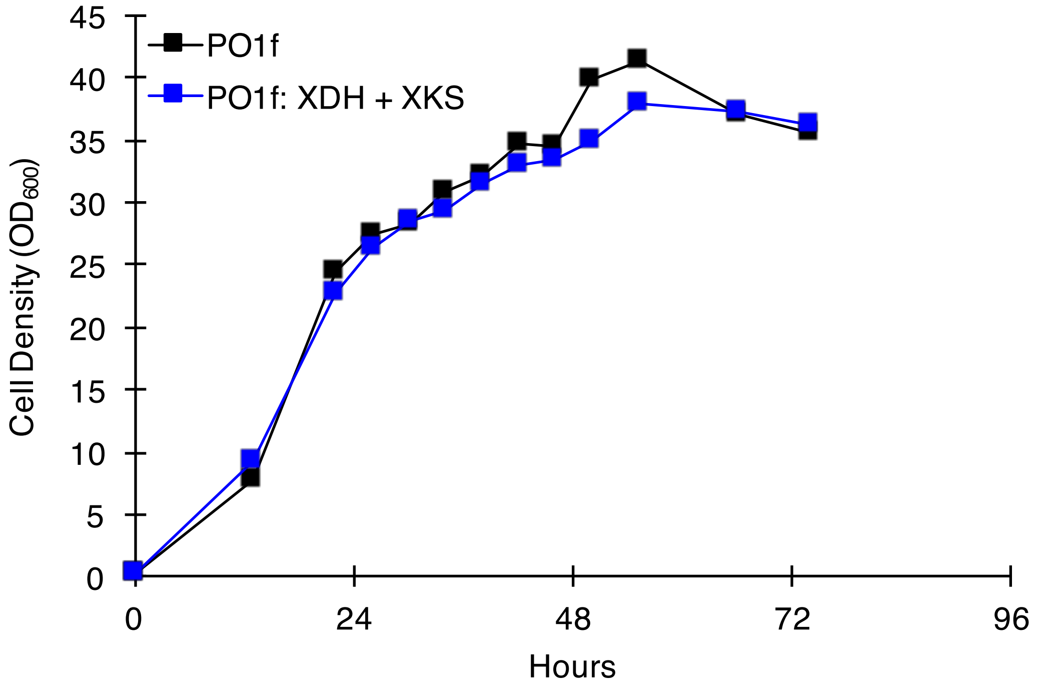

Additional File 1. Comparison of *Y. lipolytica* PO1f on Glucose.
Growth curve of *Yarrowia lipolytica* PO1f harboring either empty vectors (LEU2 and URA3) or XDH and XKS (Plamids 21 and 22).
