# Supplementary material for: Engineering xylose utilization in Yarrowia lipolytica by understanding its cryptic xylose pathway
Source: Biotechnol Biofuels. 2016 Jul 21;9:149. doi: 10.1186/s13068-016-0562-6 (PMC4955270; doi:10.1186/s13068-016-0562-6)
Supplement: Supplementary file 2 — 10.1186/s13068-016-0562-6 Candidate XYR genes in Y. lipolytica by BLAST analysis. A list of the putative XYR genes that were screened in this study, with their NCBI accession numbers and their % identity to S. stipitis XYR. [file 13068_2016_562_MOESM2_ESM.docx]

| XYR  Candidate | NCBI  Accession | BLAST (% identity)  vs XYR (*S. stipitis*) |
| --- | --- | --- |
| XYR1 | YALI0D07634g | 49 % |
| XYR2 | YALI0F18590g | 40 % |
| XYR3 | YALI0A15906g | 39 % |
| XYR4 | YALI0B21780g | 38 % |
| XYR5 | YALI0C13508g | 37 % |
| XYR6 | YALI0B07117g | 38 % |
| XYR7 | YALI0E18348g | 38 % |
| XYR8 | YALI0042092g | 37 % |
| XYR9 | YALI0C09119g | 38 % |
| XYR10 | YALI0F06974g | 37 % |
| XYR11 | YALI0B15268g | 33 % |
| XYR12 | YALI0C00319g | 33 % |
| XYR13 | YALI0A19910g | 28 % |

**Additional File 2. Candidate XYR genes in *Y. lipolytica* by BLAST Analysis**
