# Supplementary figures and images for: Engineering xylose utilization in Yarrowia lipolytica by understanding its cryptic xylose pathway
Source: Biotechnol Biofuels. 2016 Jul 21;9:149. doi: 10.1186/s13068-016-0562-6 (PMC4955270; doi:10.1186/s13068-016-0562-6)

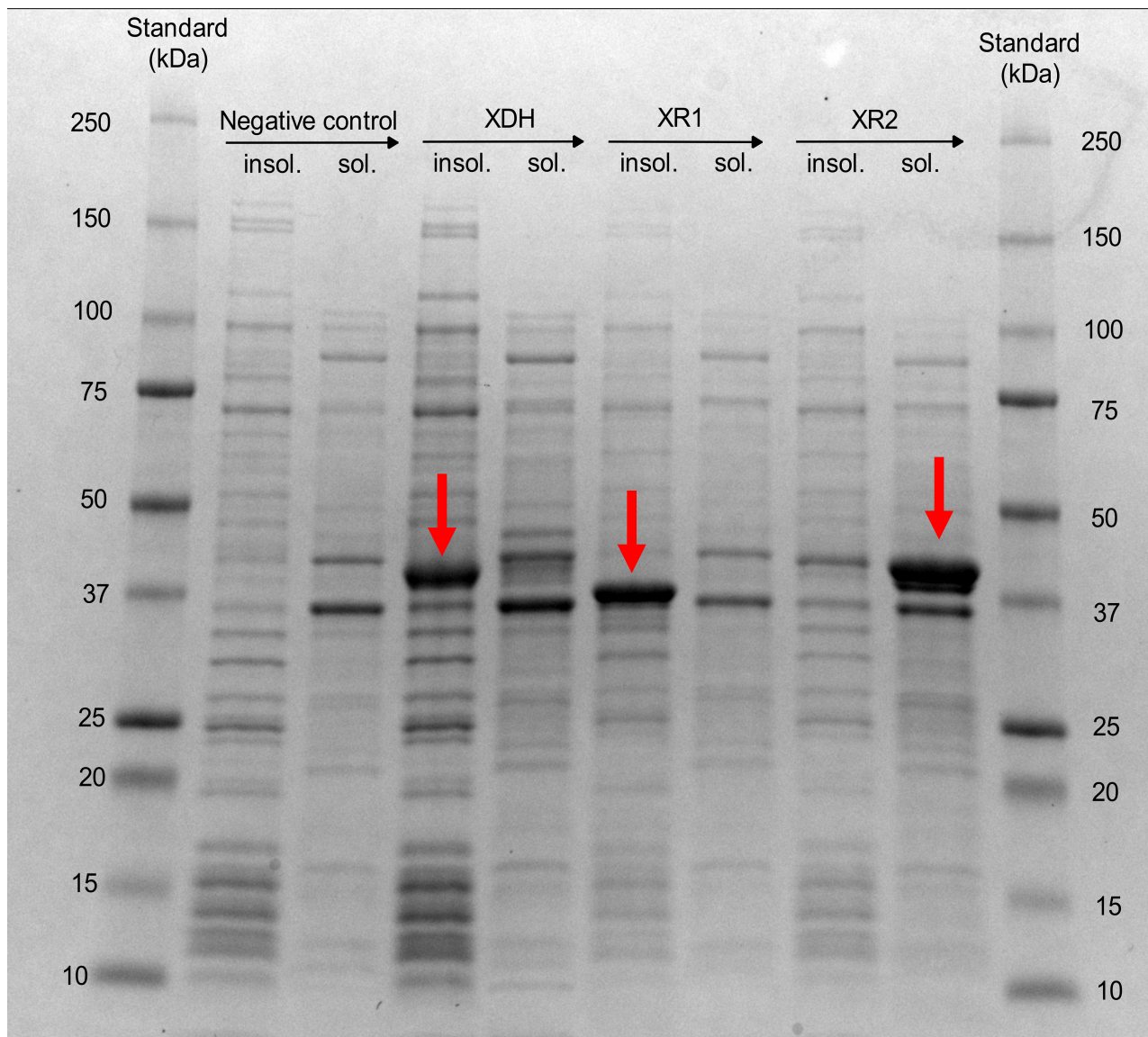

**Additional File 3. SDS-PAGE of XDH,XYR1, and XYR2 in *E. coli* BL21 lysates**

Supplement: Supplementary file 3 — 10.1186/s13068-016-0562-6 SDS-PAGE of XDH, XYR1, and XYR2 in E. coli BL21 lysates. SDS-PAGE data showing the abundance of insoluble and soluble protein from E. coli overexpression of the following enzymes: XDH, XYR1 or XYR2. [file 13068_2016_562_MOESM3_ESM.pdf]
