# Supplementary material for: Engineering xylose utilization in Yarrowia lipolytica by understanding its cryptic xylose pathway
Source: Biotechnol Biofuels. 2016 Jul 21;9:149. doi: 10.1186/s13068-016-0562-6 (PMC4955270; doi:10.1186/s13068-016-0562-6)
Supplement: Supplementary file 4 — 10.1186/s13068-016-0562-6 Oligonucleotides used in this study. A complete list of all of the oligonucleotides we used throughout this manuscript. [file 13068_2016_562_MOESM4_ESM.docx]

**Additional File 4. Oligonucleotides Used in this Study**

| **#** | **Sequence 5’ 🡪 3’** |
| --- | --- |
| 1 | TAAGGATCCAAACTCGAGTAAGGAT |
| 2 | ATGTATATCTCCTTCTTAAAAGATCTTTTG |
| 3 | TTTAAGAAGGAGATATACATATGTCCTTCAAGCTCGCCTC |
| 4 | TCTGTTTCCTCCTTACTAGTTTAGGCGAAAATGGGAAGGTTAG |
| 5 | ACTAGTAAGGAGGAAACAGAATGTCTTCTAACCCGTCATTTGTTC |
| 6 | CCTTACTCGAGTTTGGATCCTTACTACTCCTCCTCGGGACCGTCAA |
| 7 | TTTAAGAAGGAGATATACATATGTATCTCGGACTGGATCTTTCGAC |
| 8 | CCTTACTCGAGTTTGGATCCTTATTTCTCCAGGCAGGCGTTTTC |
| 9 | AAAAGATCTTTTAAGAAGGAGATATACATATGCCTTCTATTAAGTTGAACTCTGGTTAC |
| 10 | CATTCTGTTTCCTCCTTACTAGTTTAGACGAAGATAGGAATCTTGTCCCAG |
| 11 | CAAAAGATCTTTTAAGAAGGAGATATACATATGGCAGGCGGACCCACTCTCAA |
| 12 | TCTGTTTCCTCCTTACTAGTTTACTTCTTCTGCTCAGCAAGGTAC |
| 13 | CAAAAGATCTTTTAAGAAGGAGATATACATATGACTTCCATCGACTTTACCATGAAC |
| 14 | TCTGTTTCCTCCTTACTAGTTTAGCAAAAGTCAAAGTCGGGGAAAC |
| 15 | CAAAAGATCTTTTAAGAAGGAGATATACATATGACTTCTATCGACTTTACCATGAA |
| 16 | TCTGTTTCCTCCTTACTAGTTTAGCAGAAGTCGAAGTCGG |
| 17 | CAAAAGATCTTTTAAGAAGGAGATATACATATGCCAATTATCACAGAAACATTCAAG |
| 18 | TCTGTTTCCTCCTTACTAGTttaTTTGTCCTCGAAACCAAGGTTG |
| 19 | CAAAAGATCTTTTAAGAAGGAGATATACATATGTTCCGGTCAGTATATAAACG |
| 20 | TCTGTTTCCTCCTTACTAGTTTAGCAGAAGTCAAAGTCGGG |
| 21 | CAAAAGATCTTTTAAGAAGGAGATATACATATGACTTCTATCGACTTCACTATGAAC |
| 22 | TCTGTTTCCTCCTTACTAGTttaACAGAAATCAAAGTCGGGGAAAC |
| 23 | CAAAAGATCTTTTAAGAAGGAGATATACATATGACTTCTCTCGACTTTACTATGAAC |
| 24 | TCTGTTTCCTCCTTACTAGTTTAGCAGAAGTCAAAGTCGGG |
| 25 | CAAAAGATCTTTTAAGAAGGAGATATACATATGTCGCTCACTACCTCCACAA |
| 26 | TCTGTTTCCTCCTTACTAGTttaCACAGGGTATCCAAGACAGTC |
| 27 | CAAAAGATCTTTTAAGAAGGAGATATACATATGTCAATTACCGACTCATTCAAG |
| 28 | TCTGTTTCCTCCTTACTAGTttaGTCCTTAGCCCAAATGTGCTT |
| 29 | CAAAAGATCTTTTAAGAAGGAGATATACATATGACTGTGCCCAAAGTGAAAC |
| 30 | TCTGTTTCCTCCTTACTAGTTTATATTTTGGCTTCGCCCTCTT |
| 31 | CAAAAGATCTTTTAAGAAGGAGATATACATATGTCCGTTTCCATTCCTCTTC |
| 32 | TCTGTTTCCTCCTTACTAGTttaCTTGGTAACGGTGGGGTC |
| 33 | CAAAAGATCTTTTAAGAAGGAGATATACATATGGCCATTCCTACTCGAGCT |
| 34 | TCTGTTTCCTCCTTACTAGTTTAAGGAGCAACGGTGGGGT |
| 35 | CAAAAGATCTTTTAAGAAGGAGATATACATATGCCTGCACCAGCAACCTAC |
| 36 | TCTGTTTCCTCCTTACTAGTTCAAGGACAACAGTAGCCGCC |
| 37 | TATAAGAATCATTCAAAGGCGCGCCATGTATCTCGGACTGGATCTTTCGAC |
| 38 | ACATAACTAATTACATGAGGCTAGCTTATTTCTCCAGGCAGGCGTTTTC |
| 39 | TATAAGAATCATTCAAAGGCGCGCCATGTCTTCTAACCCGTCATTTGTTC |
| 40 | ACATAACTAATTACATGAGGCTAGCCTACTCCTCCTCGGGACCGTCAA |
| 41 | AATTCGAGCTCGGCGCGCCT |
| 42 | CGGATCCTGGCTGTGGTGATG |
| 43 | cCATCATCACCACAGCCAGGATCCGTCCTTCAAGCTCGCCTCCGGAAAG |
| 44 | cctgcAGGCGCGCCGAGCTCGAATTGGCGAAAATGGGAAGGTTAGCGTAG |
| 45 | cCATCATCACCACAGCCAGGATCCGTCTTCTAACCCGTCATTTGTTCTTCGAAAG |
| 46 | cctgcAGGCGCGCCGAGCTCGAATTCTACTCCTCCTCGGGACCGTCAATG |
| 47 | CATCATCACCACAGCCAGGATCCGGCAGGCGGACCCACTCTCAA |
| 48 | AGGCGCGCCGAGCTCGAATTCTTCTTCTGCTCAGCAAGGTACTTC |
| 49 | CTCACGGACTCATCAGTGGATCCGCGGTTTGAATGATTCTTATACTCAGAAG |
| 50 | GATCCACTGATGAGTCCGTGAGGACGAAACGAGTAAGCTCG |
| 51 | ATCCTCAAAGACGAGATCCAGACGAGCTTACTCGTTTCGTC |
| 52 | TCTGGATCTCGTCTTTGAGGATGTTTTAGAGCTAGAAATAGCAAGTT |
| 53 | AATTGAATTCAAAGGAGGCCATCCTATGCGAATTGGCATACCAAGAGAAC |
| 54 | CTCACTATAGGGCGAATTGGAGCTCTTACAGAGCTTTCAGGATTGCATCC |
| 55 | TACAGGAGTGTCATCCTTACGTTTTAGAGCTAGAAATAGCAAGTTA |
| 56 | GTAAGGATGACACTCCTGTAACGTCAACCTGCGCCGACCC |
| 57 | AGGACATCTTCATCACCACCAAG |
| 58 | CCACCACTCGTTCTGCTCC |
| 59 | GGAGCCACCATCTACCAGAATGAG |
| 60 | GTTCGGGCGTTGTTCCAGTTTTTG |
| 61 | CTCTGTCCGTGGCTGTCC |
| 62 | GAACAAGATCAACACACACAATGG |
| 63 | ACACGGTCACACAAGTCC |
| 64 | GACGCTCTATCAGCAAGTCC |
| 65 | ataagaatcattcaaaggcgcgccATGTCCTTCAAGCTCGCCTCc |
| 66 | acataactaattacatgaggctagcTTAGGCGAAAATGGGAAGGTTAG |
| 67 | TATAAGAATCATTCAAAGGCGCGCCATGGCAGGCGGACCCACTCTCAA |
| 68 | ACATAACTAATTACATGAGGCTAGCTTACTTCTTCTGCTCAGCAAGGTAC |
