# Supplementary material for: Engineering xylose utilization in Yarrowia lipolytica by understanding its cryptic xylose pathway
Source: Biotechnol Biofuels. 2016 Jul 21;9:149. doi: 10.1186/s13068-016-0562-6 (PMC4955270; doi:10.1186/s13068-016-0562-6)
Supplement: Supplementary file 5 — 10.1186/s13068-016-0562-6 SLIC cloning table. A complete list of all plasmids and the construction strategy we used throughout this manuscript. [file 13068_2016_562_MOESM5_ESM.docx]

**Additional File 5. SLIC Cloning Table**

|  | **Vector PCR/digest** | | | **Insert(s) PCR** | | | |
| --- | --- | --- | --- | --- | --- | --- | --- |
| **Plasmid** | **Forward**  **Oligo** | **Reverse**  **Oligo** | **Template** | **Forward**  **Oligo** | **Reverse**  **Oligo** | **Template** | **Gene of Interest** |
| Plasmid 2 | Oligo 1 | Oligo 2 | Plasmid 1 | Oligo 7 | Oligo 8 | gDNA | XKS (*Y. lipolytica*) |
| Plasmid 3 | Oligo 1 | Oligo 2 | Plasmid 1 | Oligo 5 | Oligo 6 | gDNA | XDH (*Y. lipolytica*) |
|  |  |  |  | Oligo 3 | Oligo 4 |  | XYR1 (*Y. lipolytica*) |
| Plasmid 4 | Oligo 1 | Oligo 2 | Plasmid 1 | Oligo 5 | Oligo 6 | gDNA | XDH (*Y. lipolytica*) |
|  |  |  |  | Oligo 11 | Oligo 12 |  | XYR2 (*Y. lipolytica*) |
| Plasmid 5 | Oligo 1 | Oligo 2 | Plasmid 1 | Oligo 5 | Oligo 6 | gDNA | XDH (*Y. lipolytica*) |
|  |  |  |  | Oligo 13 | Oligo 14 |  | XYR3 (*Y. lipolytica*) |
| Plasmid 6 | Oligo 1 | Oligo 2 | Plasmid 1 | Oligo 5 | Oligo 6 | gDNA | XDH (*Y. lipolytica*) |
|  |  |  |  | Oligo 15 | Oligo 16 |  | XYR4 (*Y. lipolytica*) |
| Plasmid 7 | Oligo 1 | Oligo 2 | Plasmid 1 | Oligo 5 | Oligo 6 | gDNA | XDH (*Y. lipolytica*) |
|  |  |  |  | Oligo 17 | Oligo 18 |  | XYR5 (*Y. lipolytica*) |
| Plasmid 8 | Oligo 1 | Oligo 2 | Plasmid 1 | Oligo 5 | Oligo 6 | gDNA | XDH (*Y. lipolytica*) |
|  |  |  |  | Oligo 19 | Oligo 20 |  | XYR6 (*Y. lipolytica*) |
| Plasmid 9 | Oligo 1 | Oligo 2 | Plasmid 1 | Oligo 5 | Oligo 6 | gDNA | XDH (*Y. lipolytica*) |
|  |  |  |  | Oligo 21 | Oligo 22 |  | XYR7 (*Y. lipolytica*) |
| Plasmid 10 | Oligo 1 | Oligo 2 | Plasmid 1 | Oligo 5 | Oligo 6 | gDNA | XDH (*Y. lipolytica*) |
|  |  |  |  | Oligo 23 | Oligo 24 |  | XYR8 (*Y. lipolytica*) |
| Plasmid 11 | Oligo 1 | Oligo 2 | Plasmid 1 | Oligo 5 | Oligo 6 | gDNA | XDH (*Y. lipolytica*) |
|  |  |  |  | Oligo 25 | Oligo 26 |  | XYR9 (*Y. lipolytica*) |
| Plasmid 12 | Oligo 1 | Oligo 2 | Plasmid 1 | Oligo 5 | Oligo 6 | gDNA | XDH (*Y. lipolytica*) |
|  |  |  |  | Oligo 27 | Oligo 28 |  | XYR10 (*Y. lipolytica*) |
| Plasmid 13 | Oligo 1 | Oligo 2 | Plasmid 1 | Oligo 5 | Oligo 6 | gDNA | XDH (*Y. lipolytica*) |
|  |  |  |  | Oligo 29 | Oligo 30 |  | XYR11 (*Y. lipolytica*) |
| Plasmid 14 | Oligo 1 | Oligo 2 | Plasmid 1 | Oligo 5 | Oligo 6 | gDNA | XDH (*Y. lipolytica*) |
|  |  |  |  | Oligo 31 | Oligo 32 |  | XYR12 (*Y. lipolytica*) |
| Plasmid 15 | Oligo 1 | Oligo 2 | Plasmid 1 | Oligo 5 | Oligo 6 | gDNA | XDH (*Y. lipolytica*) |
|  |  |  |  | Oligo 33 | Oligo 34 |  | XYR13 (*Y. lipolytica*) |
| Plasmid 16 | Oligo 1 | Oligo 2 | Plasmid 1 | Oligo 5 | Oligo 6 | gDNA | XDH (*Y. lipolytica*) |
|  |  |  |  | Oligo 35 | Oligo 36 |  | SDR (*Y. lipolytica*) |
| Plasmid 17 | Oligo 1 | Oligo 2 | Plasmid 1 | Oligo 5 | Oligo 6 | gDNA | XDH (*Y. lipolytica*) |
|  |  |  |  | Oligo 9 | Oligo 10 |  | XYR (*P. stipitis*) |
| Plasmid 21 | AscI | NheI | Plasmid 19 | Oligo 37 | Oligo 38 | gDNA | XKS (*Y. lipolytica*) |
| Plasmid 22 | AscI | NheI | Plasmid 20 | Oligo 39 | Oligo 40 | gDNA | XDH (*Y. lipolytica*) |
| Plasmid 23 | AatII |  | Plasmid 23 | Oligo 53 | Oligo 55 | Plasmid 25 | SCRp’-tRNAp-(XKS) |
|  |  |  |  | Oligo 54 | Oligo 56 | Plasmid 25 | (XKS)sgRNA |
| Plasmid 24 | Oligo 41 | Oligo 42 | Plasmid 28 | Oligo 45 | Oligo 46 | gDNA | XDH (*Y. lipolytica*) |
| Plasmid 25 | Oligo 41 | Oligo 42 | Plasmid 28 | Oligo 43 | Oligo 44 | gDNA | XYR1 (*Y. lipolytica*) |
| Plasmid 26 | Oligo 41 | Oligo 42 | Plasmid 28 | Oligo 47 | Oligo 48 | gDNA | XYR2 (*Y. lipolytica*) |
| Plasmid 27 | AscI | NheI | Plasmid 19 | Oligo 65 | Oligo 66 | gDNA | XYR1 (*Y. lipolytica*) |
| Plasmid 28 | AscI | NheI | Plasmid 20 | Oligo 67 | Oligo 68 | gDNA | XYR2 (*Y. lipolytica*) |
